# Evolution‐informed modeling improves outcome prediction for cancers

**DOI:** 10.1111/eva.12417

**Published:** 2016-10-21

**Authors:** Li Liu, Yung Chang, Tao Yang, David P Noren, Byron Long, Steven Kornblau, Amina Qutub, Jieping Ye

**Affiliations:** ^1^ Department of Biomedical InformaticsArizona State UniversityTempeAZUSA; ^2^ School of Life ScienceArizona State UniversityTempeAZUSA; ^3^ Department of Computer Science and EngineeringArizona State UniversityTempeAZUSA; ^4^ Department of BioengineeringRice UniversityHoustonTXUSA; ^5^ The University of Texas MD Anderson Cancer CenterHoustonTXUSA; ^6^ Department of Computational Medicine and BioinformaticsUniversity of MichiganAnn ArborMIUSA

**Keywords:** evolutionary medicine, genomics/proteomics, molecular evolution, transcriptomics

## Abstract

Despite wide applications of high‐throughput biotechnologies in cancer research, many biomarkers discovered by exploring large‐scale omics data do not provide satisfactory performance when used to predict cancer treatment outcomes. This problem is partly due to the overlooking of functional implications of molecular markers. Here, we present a novel computational method that uses evolutionary conservation as prior knowledge to discover bona fide biomarkers. Evolutionary selection at the molecular level is nature's test on functional consequences of genetic elements. By prioritizing genes that show significant statistical association and high functional impact, our new method reduces the chances of including spurious markers in the predictive model. When applied to predicting therapeutic responses for patients with acute myeloid leukemia and to predicting metastasis for patients with prostate cancers, the new method gave rise to evolution‐informed models that enjoyed low complexity and high accuracy. The identified genetic markers also have significant implications in tumor progression and embrace potential drug targets. Because evolutionary conservation can be estimated as a gene‐specific, position‐specific, or allele‐specific parameter on the nucleotide level and on the protein level, this new method can be extended to apply to miscellaneous “omics” data to accelerate biomarker discoveries.

## 1 Introduction

In the past two decades, high‐throughput biotechnologies have greatly accelerated cancer research and become an indispensable component in scientific and clinical practices. “Omics” data combined with advanced computational modeling, hold promise in discovering novel biomarkers to help improve cancer medicine (Kristensen et al., 2014). However, models constructed from global molecular profiles often consist of a large number of biomarkers that have no obvious functional relevance to the biological processes under investigation (Berger, Peng, & Singh, 2013). These biomarkers are usually selected based on statistical association, which is pestered with false‐positive results in large‐scale analysis. Inclusion of these excessive markers renders a model prone to overfitting (Cawley & Nicola, 2010; Liu et al., 2014; Ludwig & Weinstein, 2005; Sham & Purcell, 2014). In fact, biomarkers discovered by mining these “omics” data often show unsatisfactory performance when used to assist disease diagnosis, prediction of cancer outcomes, or identification of therapeutic targets (Brooks, 2012; Kulasingam, Pavlou, & Diamandis, 2010; Kwon et al., 2012; Massuti, Sanchez, Hernando‐Trancho, Karachaliou, & Rosell, 2013). Thus, many researchers advocate informed analysis that combines biological knowledge, such as functional annotations and biological pathways, with computational modeling to interpret “omics” data, hoping to identify *bona fide* biomarkers to facilitate biomedical research (Chen et al., 2009; Hill et al., 2012; McDermott et al., 2013).

Cancer is an evolutionary disease (Greaves & Maley, 2012), but cancer biomarker discovery rarely integrates evolutionary selection. Sequence conservation inferred from genomes of evolutionarily diverse species represents a valuable resource of biological knowledge. As mutations disrupting critical molecular functions have been consistently purified from the species pool over eons, sequences of functionally important genes remain conserved across species. The expression of conserved genes is also under more stringent regulation than variable genes (Liao & Zhang, 2006; Podder & Ghosh, 2010). Thus, evolutionary conservation has been used as an effective indicator of functional importance (Kumar, Dudley, Filipski, & Liu, 2011; Kumar, Sanderford, Gray, Ye, & Liu, 2012; Pei & Grishin, 2001). Evolutionary conservation has left comprehensible signatures in cancers. It has been shown that proto‐oncogenes and tumor suppressor genes are among the most highly conserved genes (Shilo & Weinberg, 1981). A majority of somatic cancer driver mutations interrupt positions that do not tolerate germline mutations (Dudley et al., 2012). Therefore, evolutionary conservation of genetic elements can provide valuable guidance to cancer biomarker discovery by eliminating spurious markers that show fortuitous statistical associations but little biological relevance.

Not all conserved genes contribute to carcinogenesis and cancer progression, and not all cancer genes are evolutionarily conserved (Ballard‐Barbash et al., 2012). Applying evolutionary conservation on cancer biomarker discovery also requires simultaneous consideration of statistical association to achieve high predictive power. In this study, we present a computational method that uses evolutionary conservation as prior knowledge within a machine learning framework to assist biomarker selection. We applied this new method to predict therapeutic responses in patients with acute myeloid leukemia (AML) and to predict metastasis in prostate cancers. The results show that evolution‐informed models enjoy high predictive accuracy using only a few functionally important biomarkers, thus ameliorated the risk of overfitting. We further show that the identified genetic markers are involved in tumor progression and embrace potential drug targets. These experiments demonstrate that evolution‐informed modeling successfully improves biomarker selection to go beyond statistical association and seek biological implications.

## 2 Materials and Methods

### 2.1 Cancer datasets

We first developed this method to participate in the DREAM 9 acute myeloid leukemia (AML) Challenge (Noren et al., 2016). A total of 31 teams from around the world, including our team, participated in this challenge. Provided by the challenge organizers and available from their official website (https://www.synapse.org/#!Synapse:syn2455683/wiki/64007), this dataset consisted of 291 patients who were newly diagnosed with AML and received induction therapy. Treatment outcomes were recorded as complete response or resistance to induction therapy. Each patient was measured on 40 clinical covariates describing demographic, cytogenic, mutation status, and the results of several standard blood tests. Proteomic data were available for each patient sample obtained prior to treatments. The proteomic features represent levels of 231 total or phosphorylated proteins, focusing on proteins involved in apoptosis, cell cycle, and signal‐transduction pathways. Seventy‐nine of these proteins have confirmed roles in oncogenesis and cancer progression (i.e., cancer driver genes), as annotated by the Cancer Gene Consensus list in the COSMIC database (Forbes et al., 2015). The goal was to predict if a patient will have a complete response or resistance to chemotherapy using clinical and proteomic markers. Of the total 291 patient samples, the DREAM organizer provided 191 samples to us for biomarker selection and model training. The other 100 samples were depleted of treatment outcome labels and used for blind testing.

The second cancer dataset was downloaded from NCBI GEO database (accession number: GSE10645). This dataset consisted of 401 patients who were diagnosed with prostate cancer and received prostatectomy (Nakagawa et al., 2008). Treatment outcomes were recorded as metastatic recurrence after surgery or no evidence of disease progression within 5 years. However, all patients have increased level of prostate‐specific antigen (PSA) that is routinely used to monitor disease recurrence. For each patient, a panel of 1,021 oncogenes, tumor suppressor genes, and genes in their associated pathways was interrogated using Agilent custom gene expression microarrays. In particular, 604 genes on this panel have previously been associated with prostate cancer progression. No clinical covariate was available for these patients. The goal was to predict metastatic recurrence using genetic markers.

### 2.2 Estimate evolutionary conservation

**Figure 1:**
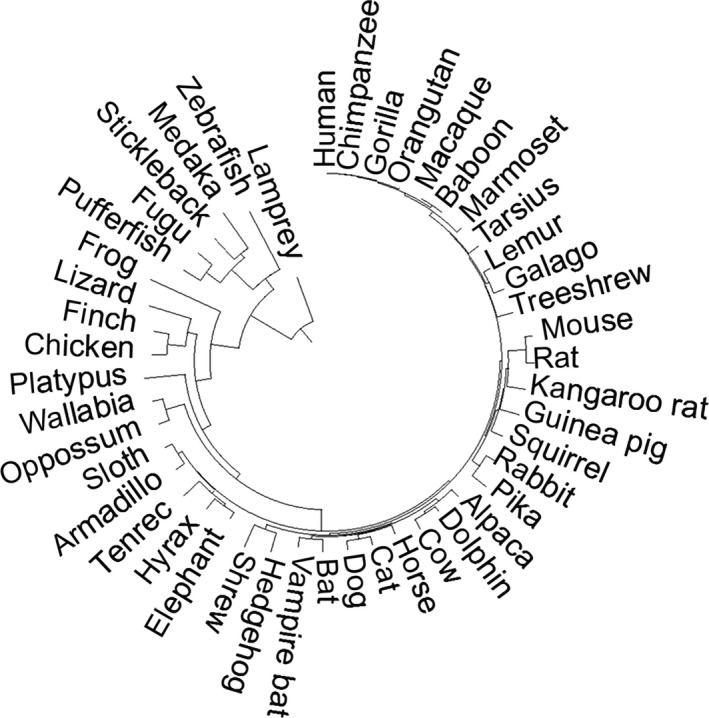
TimeTree of the 46 species used in computing evolutionary parameters. Branch length is proportional to species divergence times obtained from the TimeTree database (Hedges et al., 2006)

Using the Fitch algorithm (Kumar et al., 2012), we computed the absolute substitutional rate (*r*) of each position in a human protein sequence. Given a human protein, we retrieved multiple sequence alignments of its orthologs in 46 species available from the UCSC Genome Browser (Fujita et al., 2011). These species form a TimeTree that contains representatives from all major groups of vertebrates (Fig. 1). These species include 10 primates, 13 placental mammals, three nonplacental mammals, and nine other vertebrates that collectively represent over 500 million years of evolutionary history. The branch length between two species was set to their divergence time obtained from the TimeTree database, in the unit of million years (Hedges, Dudley, & Kumar, 2006). The total branch length of this TimeTree is 5.8 billion years. For each position in the alignments, a new tree was created containing only taxa that do not have a gap at this position. The evolutionary time span, *t* of a position equals to the sum of branch lengths in this new tree. The number of substitutions *s* is the count of different amino acids at this position. We computed absolute substitution rate *r* = 1000 × *s*/*t* in the unit of substitution/billion years. For a protein of length *L*, the evolutionary rate (*R*) was estimated as the average *r* over all positions ($R=\frac{1}{L}\sum_{i=1}^{L}r_{i}$).

### 2.3 Evolution‐informed modeling

The purpose of evolution‐informed modeling is to prioritize evolutionarily conserved and thus functionally important genes during biomarker discovery (i.e., feature selection in the machine learning field). The selected biomarkers are then used to build a predictive model (i.e., classification). It can be achieved by employing (i) a deliberately designed weighting schema, (ii) an effective feature selection algorithm, and (iii) a robust classification model.

#### 2.3.1 Composite weighting schema

Because fast evolutionary rate indicates low conservation, we used its reciprocal (1/*R*) as the evolutionary weight (*WE*). For clinical covariates, there is no meaningful score of evolutionary conservation. Because clinical features tend to have higher predictive power than molecular features in general (Falini, Nicoletti, Martelli, & Mecucci, 2007; Thiede et al., 2006; Walter, Othus, Burnett, et al., 2015; Walter, Othus, Paietta, et al., 2015), we assigned the maximum value of all WEs in the dataset to clinical features. To assess statistical significance, we performed a Student's *t* test for each feature between two clinical outcome classes (poor outcome as the positive class, good outcome as the negative class). In the presence of multiple classes, other statistical tests such as F test can be used. *p* values from these tests were transformed via negative logarithm (−log(*p*)) and used as the statistical weight (*WS*). For each feature *i*, the final weight was the sum of evolutionary weight and statistical weight (*W*_*i*_ *= WE*_*i*_ *+ WS*_*i*_). In this study, we assumed equal contribution of evolutionary conservation and statistical association to the final weights. However, their relative contributions can be adjusted based on the understanding of a specific cancer phenotype.

#### 2.3.2 Feature selection

**Figure 2:**
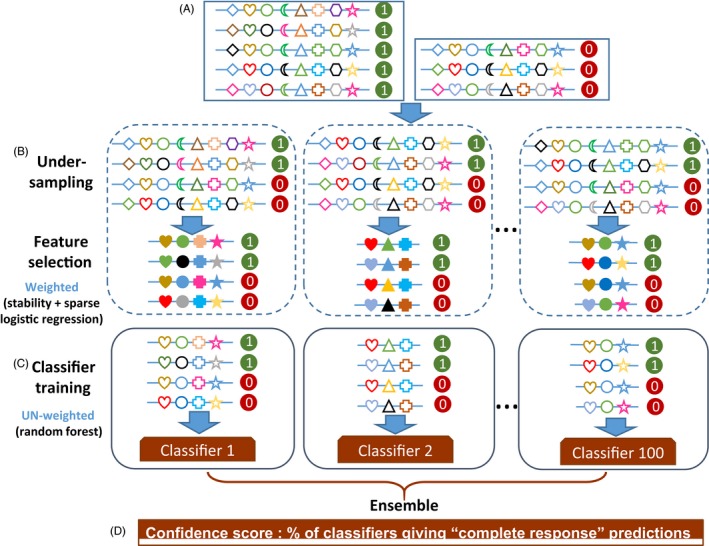
Graphical representation of the workflow of evolution‐informed modeling. (A) Input matrix. Each row represents a sample, with positive samples (i.e., with poor clinical outcomes) labeled as “1” and negative samples (i.e., with good clinical outcomes) labeled as “0.” Each column represents a feature, as indicated by different symbols. (B) Feature selection. Subsets of the input data are generated using under‐sampling that randomly chooses equal numbers of positive and negative samples. For each subset, feature values are transformed with composite weights. Feature selection is then applied on the weighted features. Using stability selection and sparse logistic regression, informative features are selected. Open symbols represent un‐weighted features. Solid symbols represent weighted features. (C) Classification model. For each subset, un‐weighted values of selected features are used to build a random forest classifier (a submodel). Collectively, these submodels comprise the ensemble model. (D) Prediction. For an unknown sample, each submodel produces a predicted label. The majority rule is used for the final prediction. The percentage of submodels that predict the sample as the positive class label is used as the confidence score of the final prediction

Within a cancer dataset, we first normalized each clinical and molecular feature by computing z‐scores that have a distribution with a mean of 0 and a standard deviation of 1. Let a feature matrix *f*_*ij*_ denote the normalized values of the *i*th features for the *j*th sample (Fig. 2A**)**. We then transformed this feature matrix by multiplying *W*_*i*_ for each feature. This weighted feature matrix *f*_*ij*_^*w*^ was subjected to feature selection (Fig. 2B). In particular, we used the *l*_*1*_‐norm regularized logistic regression, as implemented in the SLEP package (Liu, Ji, & Ye, 2009). Our purpose is to solve the following problem:

(1) $$min_{x}\;\sum_{j=1}^{m}\;\log\left(1+\exp\left(-y_{j}\left(x^{T}\;f_{j}^{w}+c\right)\right)\right)+{\lambda ||x||}_{1}$$

where *y*_*j*_ and *f*_*j*_^*w*^ are the class label and the weighted feature vector for the *j*th sample, respectively, *c* is a constant corresponding to the intercept in a linear model, λ is the regularization parameter, and *x* is the solution. By assigning higher weights to evolutionarily conserved and/or statistically significant features, we increased the absolute value of *f*_*j*_^*w*^. In fact, the formulation is (1) is equivalent to the following problem:

(2) $$min_{x}\;\sum_{j=1}^{m}\;\log\left(1+\exp\left(-y_{j}\left(x^{T}\;f_{j}+c\right)\right)\right)+\;\lambda \sum_{i}\frac{1}{W_{i}}\;\left|x_{i}\right|$$

In equation (2), a larger penalty is imposed on features with a small weight. Consequently, the solution will favor the features with a large weight.

In equation (1) and (2), the calculation of *x* requires the selection of the most appropriate regularization parameter (λ), which dictates the number of features selected (receiving nonzero *x* values). To reduce such dependence, we employed a stability selection method. In particular, 100 bootstraps were performed to identify features that are consistently selected in more than 50% of runs of the algorithm with different λ values.

#### 2.3.3 Classification

A classification model was constructed with selected features (Fig. 2C). In this step, the unweighted feature matrix *f*_*ij*_ was used to avoid biases. The classification model was a random forest with 50 trees, as implemented in the TreeBagger function in Matlab (version R2013a). While we chose to use random forest for classification, other linear or nonlinear algorithms can be employed as well.

#### 2.3.4 Bootstrapping

To avoid bias caused by the imbalance of class size (García, Sánchez, Mollineda, Alejo, & Sotoca, 2007), we wrapped a bootstrapping process around the above feature selection and classification steps. Specifically, a subset of equal numbers of samples was randomly selected from each class. This number was determined as 90% of samples in the under‐represented class. For each bootstrap, a classification model was obtained, which is called a submodel. By repeating this procedure 100 times, an ensemble of 100 submodels were produced.

#### 2.3.5 Prediction

To classify an unknown sample, 100 predictions were made, one from each submodel. The final prediction was derived by computing a confidence score, which equals to the percentage of submodels that predict the sample as the positive class label (Fig. 2D).

#### 2.3.6 Performance evaluation

We used balanced accuracy (BAC, defined as the average of true‐positive rate and true‐negative rate) and area under the receiver operating characteristic (AUROC) to assess the predictive accuracy of a model. These two parameters are robust to the imbalance of class size, and thus commonly used and well documented (García et al., 2007; Noren et al., 2016).

## 3 Results

### 3.1 Predict therapeutic responses in acute myeloid leukemia

**Figure 3:**
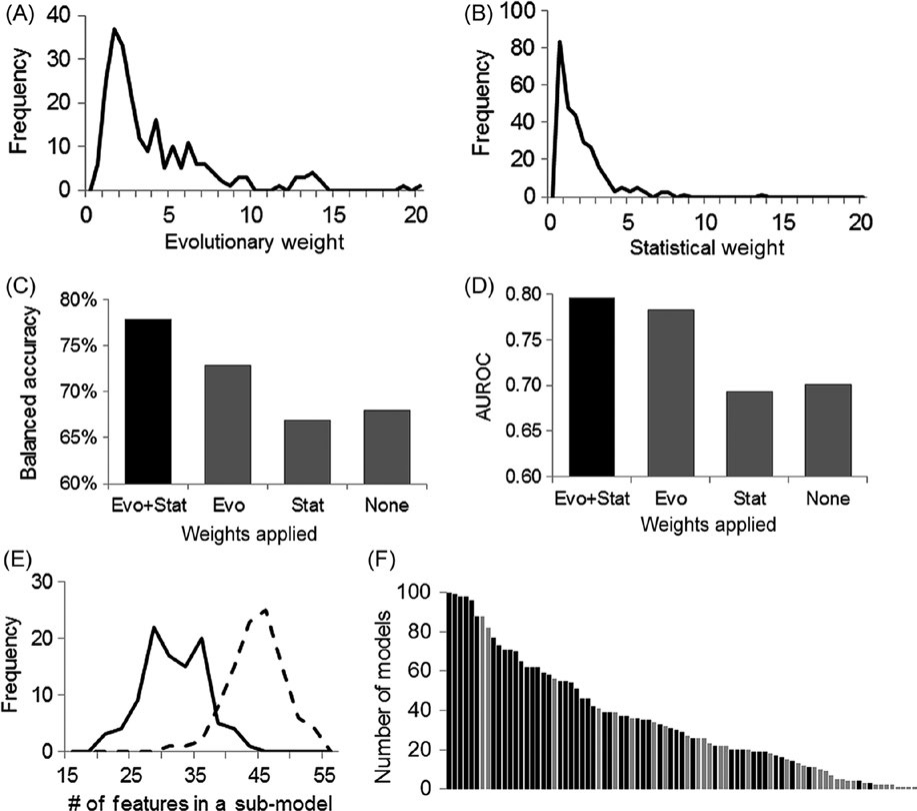
Evolution‐informed modeling to predict treatment outcomes for AML patients. Distributions of evolutionary weights (A) and statistical weights (B). Balanced accuracy (C) and AUROC (D) value of models that uses composite weight, only evolutionary weight, only statistical weight and no weight. (E) Distribution of the number of features in each submodel when composite weight (solid line) or no weight is used (broken line). Number of features is an indicator of the complexity of a model. (F) Number of submodels in which a clinical feature (black bars) or a proteomic feature (gray bars) is included. Plot consists of 85 features that were included in at least one submodel when composite weight is used

We first examined the distributions of evolutionary weights and statistical weights in the AML training dataset that consisted of 191 patient samples. Both showed left skewness (Fig. 3A,B), indicating that most proteins were not functionally critical and not statistically associated with the treatment outcome. Therefore, only a small number of biomarkers were present (i.e., sparse solution). We then applied the new method to build an evolution‐informed model. When evaluated on the held‐out testing samples that consisted of 100 unseen patient samples, our evolution‐informed model achieved the highest performance among a total of 31 participating teams from around the world, with balanced accuracy of 77.9% and AUROC of 0.796. The runner‐up had a slightly lower AUROC (0.783) but much lower balanced accuracy (72.8%) (Noren et al., 2016).

To further understand the impact of evolutionary weighting on feature selection and classification accuracy, we compared four different models (M_e+s_, M_e_, M_s,_ and M_0_), in which composite weight, only evolutionary weight, only statistical weight or no weight was used during feature selection, respectively. The rest of the algorithm was kept the same. Our results showed that M_e+s_ achieved the highest performance, with up to 11.0% increase on balanced accuracy and 0.102 increase on AUROC as compared to other models (Fig. 3C,D). Interestingly, M_s_ that used only statistical weight showed the lowest performance. In an effort to understand this, we split the training dataset into two random subsets and performed a Student's *t* test within each subset. The correlation of *p*‐values between these two subsets was only moderate (coefficient = .37), reflecting high noise level in proteomic data. Therefore, algorithms that solely rely on statistical associations to choose biomarkers from “omics” data may suffer from over‐fitting, as reported by other studies as well (Liu et al., 2014). Evolutionary information, as demonstrated in our method, can help effectively reduce the noise level and prioritize genes that are biologically important.

Applying weights during feature selection also helped reduce the complexity of the model, as measured by the number of features included in each submodel (Fig. 3E). In the feature selection step, features that were selected in >50% bootstrapping runs with a wide range of regularization parameters are regarded as important and informative. Under this setting, M_e+s_ achieved an accuracy of 77.9% with an average of 30 features in each submodel. Contrarily, in M_0_, the accuracy dropped to 68% and the average number of features increased to 43 in each submodel. These excessive features are likely false‐positive markers. The fact of significantly fewer features achieving significantly higher accuracy demonstrates the power of using evolutionary and statistical weights to assist feature selection and classification for predicting AML outcomes.

Several studies showed that clinical features were more informative than proteomic features in predicting AML outcomes (Cilloni et al., 2008; Gulley, Shea, & Fedoriw, 2010; Moon et al., 2010; Noren et al., 2016), which was also reflected in our model. Among the most frequently used features that were included in more than 80% of submodels, only two are proteomic (Fig. 3F, Table S1). However, these two proteins, namely PIK3CA and GSK3, both have strong implications in AML therapies. PIK3CA is a well‐known proto‐oncogene (Zhu et al., 2012). The PIK3CA signaling pathway is a drug target in treating several hematologic malignancies (Jabbour, Ottmann, Deininger, & Hochhaus, 2014). GSK3 plays a role in the control of several regulatory proteins including the proto‐oncogene JUN, and in the WNT and PI3K signaling pathways that are critical in tumor progression. Recently, GSK3A has been suggested as a potential target for treating AML (Banerji et al., 2012). Selection of these two potential drug targets without knowing such information in prior demonstrated that evolution‐informed modeling is capable of identifying biomarkers that are computationally powerful and biologically meaningful as well. It is also worth noting that both PIK3CA and GSK3 are conserved proteins although they are not the most conserved ones in this assay. Similarly, their statistical associations are significant but not among the top ones either. Therefore, evolutionary and statistical weights do not over‐dominate the selection of features. This gave us the desired effect on the feature selection process, in which functional importance and statistical significance are emphasized, but other factors, such as minimization of classification errors, still play essential roles.

### 3.2 Predict metastasis in prostate cancer

**Figure 4:**
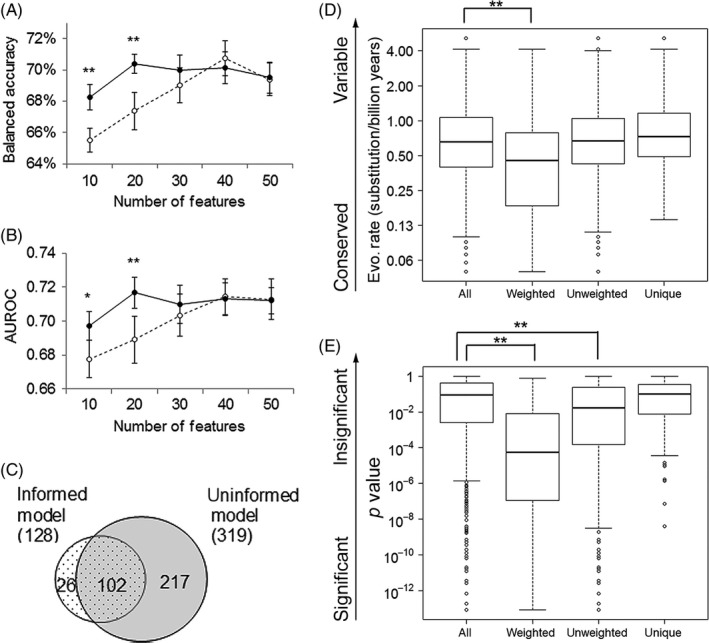
Evolution‐informed modeling to predict metastasis for prostate cancers. Balanced accuracy (A) and AUROC values (B) for evolution‐informed models (solid lines) and for un‐weighted models (broken lines) that include various numbers of features. Average values with standard errors are plotted. * and ** indicate significant difference with *t* test *p* value <.05 or <.01, respectively. (C) Venn diagram of proteins included in the top‐performing evolution‐informed model and in the top‐performing uninformed model. Box plots to compare the distributions of evolutionary rate (D) and statistical significance (E) between all proteins, proteins included in the top‐performing evolution‐informed model, proteins included in the top‐performing uninformed models, and proteins unique to the top‐performing uninformed model. ** indicates significant difference with *t* test *p* value <.01

In this study, we applied the evolution‐informed modeling and evaluated its performance by followed a strict cross‐validation procedure. Specifically, we randomly chose 80% of the samples for training and used the other 20% for independent testing. This procedure was repeated 10 times, and the averages of balanced accuracy and AUROC values estimated from the test datasets were reported. For each iteration, we built an M_e+s_ model that incorporated evolutionary and statistical weights, and an M_0_ model that did not employ any weight. In addition to finding the optimal model with the highest prediction accuracy, this dataset allowed us to examine the performance of models with varying complexity, as measured by numbers of features included. We found that M_e+s_ achieved the largest improvement over M_0_ when the models are the simplest (Fig. 4A,B). If only 10–20 genes were allowed for each submodel, M_e+s_ had 4% higher accuracy (paired *t*(19) = 4.95, *p *=* *4.5 × 10^−5^) and 4% higher AUROC values (paired *t*(19) = 4.08, *p *=* *3 × 10^−3^) than M_0_. The improvement became insignificant when the complexity of a model increased and reached 40 genes in each submodel. While the best performance of M_e+s_ is similar to that of M_0_ (balanced accuracy: 70.8% vs. 70.1%, AUROC: 0.721 vs. 0.731), M_0_ used twice as many features as M_e+s_ (number of features included in each submodel: 40 vs. 20).

We further examined genes used in models with the best performance. Summarized over all submodels, 128 and 319 unique genes were included in at least one submodel in the top‐performing M_e+s_ model and in the top‐performing M_0_ model, respectively. Most genes (80%) in M_e+s_ were also present in M_0_, while M_0_ contained 217 additional genes (Fig. 4C). Compared to all genes assayed, these additional genes are less conserved (*t* test on log(evolutionary rate), *t*(396) = 3.11, *p *=* *.002, Fig. 4D) and have weaker statistical associations (*t* test on log(*p* value), *t*(499) = 3.30, *p *=* *.001, Fig. 4E). Because including them in the predictive models negatively affected the accuracy, they are probably irrelevant to the metastasis phenotype. Indeed, GeneOntology analysis (Mi et al., 2016) showed that these additional genes are not enriched in any biological process. Contrarily, genes in M_e+s_ have higher conservation (*t* test on log(evolutionary rate), *t*(150) = −5.33, *p *= 10^−7^) and stronger statistical association (*t* test on log(*p* value), *t*(144) = −10.1, *p *= 10^−18^). They are significantly enriched in biological processes that have been previously implicated in metastasis and tumor progression, such as DNA repair, cell cycle, and DNA metabolism (Table S2).

## 4 Discussion

As one of the leading causes of morbidity and mortality in the modern world, cancer has become a major problem in public health. Accurate prediction of a patient's response to treatment and prognosis can greatly assist clinicians to choose appropriate therapy and help improve patient care. High‐throughput biotechnologies have generated a large amount of “omics” data that can be used for this purpose. However, the high noise level in these data impairs the usage in identifying reliable biomarkers. Further, the number of samples tested in an “omics” study is usually several orders of magnitudes smaller than the number of molecular features measured, which makes traditionally derived statistical models prone to overfitting. In fact, our analysis showed that statistical scores tended to describe random error or noise instead of the true underlying relationship in omics data. Consequently, these models are hard to interpret and lack generalization capability.

To reduce the noise, we studied the possibility of using evolutionary conservation to prioritize functionally important genes as predictive biomarkers. Evolutionary selection at the molecular level is nature's test on functional impact of genetic elements (Kimura, 1983). Compared to other functional annotations, such as functional domains and pathways that vary across tissue and developmental stages, sequence conservation is directly associated with functional consequence and rigorously tested over eons of evolutionary history (Pei & Grishin, 2001). In this study, we developed a mathematical framework that favorably includes conserved genes for biomarker discovery. By applying this new method to predict treatment outcomes for a hematological cancer (AML) and for a solid tumor (prostate cancer), we demonstrated that evolution‐informed models indeed improved the prediction accuracy on cancer outcomes. This helps eliminate irrelevant features that are often included due to stochastic factors. Thus, more reliable biological inferences can be made using features selected in the evolution‐informed procedure.

Gene expression profiles and protein expression profiles modeled in this study are molecular changes downstream of genomic alterations. Genomic aberrations play critical roles in carcinogenesis and fuel tumor heterogeneity in and between patients. Such high molecular heterogeneity forms the foundation of diverse clinical outcomes and other cancer phenotypes, as well as makes hunting of cancer driver mutations very challenging (Heng, 2015). Our previous study showed that frequently observed cancer mutations are enriched at evolutionarily conserved positions (Dudley et al., 2012). Thus, evolutionary conservation estimated at the nucleotide level may help prioritize cancer driver mutations. This suggests that genomic profiles, transcriptomic profiles and proteomic profiles of patients with cancer can be integrated and prioritized simultaneously under a common evolutionary framework.

Another aspect of cancer evolution is subclonal evolution within a tumor (Greaves & Maley, 2012). An increasing number of studies have reported that drug resistance and disease relapse in various types of cancers are attributed to expansion of preexisting or newly emerged subclones (Burrell & Swanton, 2014; Ding et al., 2012; Landau, Carter, Getz, & Wu, 2014). Given the highly dynamic characteristic of subclones, similar challenges exist in identifying driver subclones as in identifying driver mutations. As cancer is a disease of evolution that accumulates genetic mutations while it progresses, it is attractive to use mutational load to prioritize subclones. However, we may also argue that functional impact of a subclone is more informative than mutational load. In this sense, species‐level evolutionary conservation can be used to derive a composite weight that represents aggregated functional impact of all mutations in a subclone. Integrating evolutionary signatures on species level and on individual level would be a promising and exciting new direction of research.

Meanwhile, biomarker discovery shall not leave out clinical covariates that have been associated with cancer treatment outcomes in numerous studies. One difficulty we encountered in incorporating clinical covariates in evolution‐informed modeling was the calculation of meaningful and distinctive priorities for them. Currently, we rely on statistical weights computed from the training data, which do not reflect the rich domain knowledge. In the future, we will consider deriving scores from meta‐analysis, which may serve as a better surrogate of prior knowledge aggregated from existing studies. By integrating multisource omics data and clinical features and comparing evolutionary contributions and statistical contributions to clinical outcomes, we will gain new insights into the causes of cancer formation and progression.

## Data Archiving Statement

This study was conducted using publicly available data that can be downloaded from the DREAM Challenge website and the Gene Expression Omnibus database.
