# Supplementary material for: Evolution‐informed modeling improves outcome prediction for cancers
Source: Evol Appl. 2016 Oct 21;10(1):68–76. doi: 10.1111/eva.12417 (PMC5192825; doi:10.1111/eva.12417)
Supplement: Supplementary file 1 [file EVA-10-68-s001.docx]

Supplementary Table 1. Features included in more than 80% of sub-models for AML prediction.

| # of Sub-models | Features | Category | WE | WS |
| --- | --- | --- | --- | --- |
| 100 | treatment administered (Flu-HDAC) | Clinical | NA | 13.2 (1%) |
| 99 | cytogenic category (21) | Clinical | NA | 1.8 (32%) |
| 98 | level of CD34 marker detected | Clinical | NA | 4.6 (4%) |
| 98 | cytogenic category (-5) | Clinical | NA | 1.9 (28%) |
| 96 | age at diagnosis | Clinical | NA | 8 (1%) |
| 88 | Total myeloid blast cells in blood | Clinical | NA | 1.9 (29%) |
| 88 | PIK3CA | Proteomic | 9.0 (8%) | 7.1 (2%) |
| 82 | GSK3A_B.pS21* | Proteomic | 13.5 (2%) | 2.5 (19%) |

*GSK3A or GSK3B genes with Serine at position 21 phosphorylated.

Supplementary Table 2. GeneOntology (GO) analysis of 128 genes in the top-performing evolution-informed models for prostate cancers. Analysis was conducted using the PANTHER web tool ([Mi et al. 2016](#_ENREF_35)). False discovery rate (FDR) was computed using the Benjamini-Hochberg procedure ([Benjamini and Hochberg 1995](#_ENREF_3)).

| GO Term | Fold of  Enrichment / Depletion | FDR |
| --- | --- | --- |
| blood coagulation | 2.53 | 3.27E-02 |
| protein metabolic process | 0.62 | 3.28E-02 |
| response to external stimulus | 2.38 | 4.19E-02 |
| protein phosphorylation | 0.44 | 4.76E-02 |
| cell death | 0.61 | 6.38E-02 |
| apoptotic process | 0.61 | 6.38E-02 |
| DNA metabolic process | 1.65 | 7.18E-02 |
| cellular amino acid metabolic process | 0.2 | 7.83E-02 |
| cell communication | 0.81 | 7.87E-02 |
| induction of apoptosis | 0.36 | 8.45E-02 |
| cell-matrix adhesion | 2.89 | 8.68E-02 |
